# Integration of Digital Tools Into Community Mental Health Care Settings That Serve Young People: Focus Group Study

**DOI:** 10.2196/27379

**Published:** 2021-08-19

**Authors:** Ashley A Knapp, Katherine Cohen, Jennifer Nicholas, David C Mohr, Andrew D Carlo, Joshua J Skerl, Emily G Lattie

**Affiliations:** 1 Feinberg School of Medicine Northwestern University Chicago, IL United States; 2 Orygen Centre for Youth Mental Health University of Melbourne Melbourne Australia; 3 Ohio Guidestone Organization Berea, OH United States

**Keywords:** digital mental health, treatment, young people, children, adolescents, community mental health care, mobile phone

## Abstract

**Background:**

Digital mental health tools have substantial potential to be easily integrated into people’s lives and fundamentally impact public health. Such tools can extend the reach and maximize the impact of mental health interventions. Before implementing digital tools in new settings, it is critical to understand what is important to organizations and individuals who will implement and use these tools. Given that young people are highly familiar with technology and many mental health concerns emerge in childhood and adolescence, it is especially crucial to understand how digital tools can be integrated into settings that serve young people.

**Objective:**

This study aims to learn about considerations and perspectives of community behavioral health care providers on incorporating digital tools into their clinical care for children and adolescents.

**Methods:**

Data were analyzed from 5 focus groups conducted with clinicians (n=37) who work with young people at a large community service organization in the United States. This organization provides care to more than 27,000 people annually, most of whom are of low socioeconomic status. The transcripts were coded using thematic analysis.

**Results:**

Clinicians first provided insight into the digital tools they were currently using in their treatment sessions with young people, such as web-based videos and mood-tracking apps. They explained that their main goals in using these tools were to help young people build skills, facilitate learning, and monitor symptoms. Benefits were expressed, such as engagement of adolescents in treatment, along with potential challenges (eg, accessibility and limited content) and developmental considerations (eg, digital devices getting taken away as punishment). Clinicians discussed their desire for a centralized digital platform that securely connects the clinician, young person, and caregivers. Finally, they offered several considerations for integrating digital tools into mental health care, such as setting up expectations with clients and the importance of human support.

**Conclusions:**

Young people have unique considerations related to complex accessibility patterns and technology expectations that may not be observed when adults are the intended users of mental health technologies. Therefore, these findings provide critical insights to inform the development of future tools, specifically regarding connectivity, conditional restraints (eg, devices taken away as punishment and school restrictions), expectations of users from different generations, and the blended nature in which digital tools can support young people.

## Introduction

### Background

As digital devices become increasingly ubiquitous, there is growing potential for digital mental health (DMH) tools to be easily integrated into people’s daily lives and have a fundamental public health impact. DMH tools may maximize the public health impact of mental health support and extend its reach to new populations while also enabling unprecedented individualization and treatment optimization. However, efficacious DMH interventions have not yet been translated into effective and sustained use in real-world care settings. This well-described concern is known as the research-to-practice gap [1,2]. Furthermore, engagement with DMH tools is low, for example, Baumel et al [3] report that just 4% of mental health apps are opened daily. These engagement and implementation challenges may arise because conditions (eg, needs, preferences, goals, and barriers) are specific to deployment settings, and stakeholders have not traditionally been considered in the design of evidence-based DMH care.

Recently, the DMH field has attempted to address the research-to-practice gap by focusing on engagement and implementation. Frameworks, such as the Accelerated Creation-to-Sustainment (ACTS) model [4], have been proposed to guide the successful implementation and sustainment of tools in real-world settings. The ACTS model comprises three iterative stages (Create, Trial, and Sustainment) and encourages evaluation and design at each stage. For example, in the *Create* stage, the framework encourages in-depth qualitative assessments (eg, interviews and design workshops) and usability testing to inform the design of the service and its implementation alongside the technology. In addition, ACTS and other frameworks focused on the swift translation of research evidence to practice, such as the Designing for Accelerated Translation [5], recommend the incorporation of user-centered design methods throughout all phases of development to better identify user needs and context, as well as relevant surrounding factors of deployment settings. These methods intend to partner with key stakeholders to better understand current needs and existing organizational structures in an effort to design targeted, pragmatic, and sustainable DMH tools that can be optimally integrated into the proposed context of use.

A recent review highlighted the variety of factors that may impact health care providers' willingness to adopt digital health tools, including technological aspects such as ease of use, compatibility, and personalization and social and organizational factors, including workflow, evidence base, and monetary considerations [6]. However, specifically with regard to mental health, research to understand the context, needs, and goals of providers’ use of DMH tools in clinical care settings is sparse. This is especially true for child and adolescent mental health providers, despite indications that the potential for success of technological tools within clinical care may be highest for young clients, given this generation’s immersion and interest in technology [7,8]. Furthermore, young people with mental health difficulties are a critical population, as most mental health conditions emerge in adolescence and early adulthood, underscoring the importance of preventive and early intervention efforts [9-11]. However, very few studies have specifically reported on the implementation and engagement considerations of DMH tools among mental health workers who primarily work with young people [12-14].

Through a quantitative survey, Cliffe et al [12] assessed child mental health professionals’ use of and attitudes toward technology in clinical care. They found that most clinicians were uncertain of which technologies were available, which led to their primary use of older technologies, such as helplines and websites. Although not as frequent, clinicians reported the use of apps focused on emotional management and mindfulness. Similarly, Orlowski et al [13] used focus groups and semistructured interviews with mental health workers serving young people to explore the acceptability of technology to engage with clients. Clinicians identified potential strengths of technology, such as tracking symptoms or increasing engagement, but expressed concerns about internet access in rural areas, maintaining confidentiality, and crisis management. To the best of the authors’ knowledge, the only other published study that elicited feedback from mental health workers serving young people was specific to the use of a single app that asked young people to chart their symptoms as an augment to their treatment sessions [14]. Some key takeaways from this exploratory study were that clinicians found the mood graphs were helpful in engaging parents in their child’s treatment and that privacy, anonymity, and connectivity were important to the success and use of this app.

### Objectives

Given the importance of understanding the nuances in providing care to young people in efforts to successfully integrate DMH tools into health services for this population, additional data to build on early exploratory studies are needed. By focusing on unique stakeholder needs and contextual factors of different care settings, we can begin to cultivate a body of literature that could serve as a guide in informing the design and testing of technological tools for mental health workers who serve young people. In turn, we may be able to create tools that are effective, pragmatic, and sustainably integrated into care settings. This study aims to extend the literature on how mental health clinicians are currently using technology within their clinical care to young people and expand upon previous work by investigating the challenges of technology use in treating this critical population. In addition, we explore the types of digital tools that clinicians find helpful in their support of young people and considerations regarding integrating these tools into everyday practice.

## Methods

### Participants

We used data collected from clinical staff members who provide behavioral health care for children and adolescents at a large community service organization in a Midwestern state. A partnership was formed after the organization reached out to our research center and indicated an interest in incorporating technology into their behavioral health care services.

### Procedures

Focus groups were divided based on the clinical services provided by the staff members and lasted approximately 1 hour. There were 7 groups in total; 2 comprised clinicians who provide in-home services for children and adolescents (labeled as *in home 1* and *in home 2*), two comprised those who provide in-school services for children and adolescents (labeled as *school 1* and *school 2*), one comprised those who provide services through an outpatient clinic (labeled as *outpatient 1*), one comprised those who provide services to adults, and one comprised clinical supervisors. As the focus of this study was on the perspectives of clinicians serving young clients, the data from the groups comprising clinicians serving adults only and the group of clinical supervisors were excluded from analyses; thus, in total, 5 focus groups were used for this study.

Participants were recruited through email. The focus groups took place in conference rooms at the organization's central office. Groups were run by an academic research clinical psychologist and the director of our center's research operations, who has a master’s degree in public health and a background in community mental health. The focus groups were recorded and transcribed with the participants’ consent. All participants who took part in the focus groups were given US $5 gift cards and were provided with food during the group. For the clinician groups, participants were first asked questions regarding their typical clinical encounters, client communication, use of supplemental treatment resources or tools, and interest in technology-based resources or tools. They were then asked questions regarding a specific technology-based tool that has been validated in several previous studies (IntelliCare) [15-17]. Clinicians were asked what would be needed to implement this tool and similar DMH tools and how they might fit in with their current practices, which led to a broader discussion on desired DMH digital tools, features, and integration considerations. All study procedures were approved by the authors’ institutional review board before enrolling the participants.

### Data Analysis

#### Overview

This study generated an extensive mixed methods data set that provided insight into several questions related to how technology-enabled mental health interventions could be used in community mental health care. Because of the size and richness of the data set, we conducted two sets of analyses. The first used mixed methods (including quantitative measures of implementation climate and clinical orientation in conjunction with focus groups) to understand community mental health providers and supervisor attitudes toward using a variety of technologies in their work and identify barriers to and facilitators of implementation [18]. In contrast, this study is a purely qualitative analysis of issues specific to the use of technology-enabled mental health services with young clients from the perspective of child and adolescent clinicians only, as mental health treatment with young people presents unique challenges and innovation in youth community mental health care remains understudied [12,13,19]. Code creation, codebook formation, coding, and derived themes were independently performed for each analysis. The procedure for this study is outlined below.

#### Thematic Coding

Focus group transcripts were coded by authors using a thematic analysis approach [20]. Coders first reviewed the transcripts for thematic content and created a codebook with the primary themes they identified. After the codebook was created, 2 coders reviewed the transcripts a second time to ensure the codebook accuracy before completing a final round of coding. The team-based approach to coding allowed for analyst triangulation, providing a check for validity and rigor within the analysis. Within this process, themes at every step of the analysis and reporting were determined by consensus among researchers [21]. In an additional verification of the coding of the data and implications derived, the research team partnered with a key stakeholder from the community behavioral health care organization to verify the credibility of the results and implications drawn. In addition, author KC, who was independent of the coding process, compared the results with those of other analyses to verify that there was no overlap between papers. The research question explored in this paper expands on the results discussed in the previously published analysis [18], and the results of that analysis are presented to provide the necessary context.

## Results

### Participants

The community service organization serves approximately 27,000 people annually and has offices and clinics across the state serving several different local communities, with most serving low socioeconomic status. The organization serves a broad range of behavioral health concerns in youth, ranging from issues such as attention-deficit/hyperactivity disorder to depression. The clinicians in the focus groups reported that they serve youth with a wide range of presenting concerns. In addition, 61.5% (218/354) of the staff at this organization had a master’s degree, 87.0% (308/354) were female, and 72.5% (257/354) were White. A total of 37 staff members participated in the 5 focus groups included in this analysis (7 in home 1, 7 in home 2, 10 in school 1, 7 in school 2, and 6 in outpatient 1).

### Overview of Themes

Clinicians’ feedback regarding the opportunities, challenges, and future directions for integrating digital tools into clinical care with youth was divided into three main themes. Theme 1 identifies the potential strengths of digital tools, highlighting how digital tools can help young people build skills, facilitate learning, and monitor symptoms. Theme 2 presents the potential challenges of using digital tools in practice, such as limited accessibility, outdated programs, and limited content. Theme 3 introduces clinicians’ ideas for future DMH work, including their desire for a centralized digital platform and considerations for integration into care. Table 1 summarizes the key findings across the themes.

**Table 1:** Summary of key findings.

Themes and subthemes			Key findings
**Theme 1: Digital tools in clinical care with young people**			
	Build skills, facilitate learning, and monitor symptoms	Clinicians reported using digital platforms was an excellent way to better engage young people in treatment. There was a notable variety in the types of tools that clinicians used, and the main goals of using these tools were to acquire and practice skills, facilitate learning and discussion, and monitor symptoms. An example was the use of mood-tracking apps to facilitate conversations around patterns and precursors to changes in young people’s mental health symptoms. Clinicians noted the importance of considering the child’s developmental stage when deciding their level of involvement in digital tools.	
**Theme 2: Challenges of using digital tools in practice**			
	Accessibility	Challenges related to young people’s limited or no access to broadband and/or digital devices outside of treatment sessions were frequently mentioned, expanding the definition of accessibility. Clinicians mentioned other restrictions caregivers and schools placed on smartphone use, such as limits on screen time and the context in which the device can or cannot be used. These restrictions, although typically implemented by parents to promote well-being, can significantly interfere with the ability of adolescents to engage in DMH^a^ programs at times when they perceive themselves most in need of the tools. As for connectivity and smartphone access, our results imply that solutions, such as making content available offline or providing desktop versions, should be considered so that accessibility is not dependent on consistent internet and smartphone access.	
	Outdated programs and limited content	Although clinicians could see the utility of digital platforms beyond outdated designs, young people were often deterred from initially engaging or maintaining sustained use with these platforms based on the outdated design. Similarly, other digital tools clinicians used had limited content, and thus their clients would become bored quickly or tire easily of these apps because they ran out of new content quickly. To combat the vast drop-off observed in these instances, clinicians underscored the importance of keeping the design and content of young people–facing platforms fresh and up to date.	
**Theme 3: Desired digital platform and integration considerations**			
	Centralized digital platform	Several clinicians desired a centralized digital tool that securely connected the clinician, the young person, and the young person’s family. Clinicians were especially drawn to the possibility of this tool to help generalize skills learned in sessions to young people’s day-to-day lives, track mood patterns in real time to discuss in treatment, and increase parent communication and engagement in their child’s treatment. For this to be effective, clinicians underscored the importance of building rapport and attaining buy-in from parents and other stakeholders so that teens have access to devices and services to use as therapy resources when triggered at school or home.	
	Considerations for integration into care	Regarding the design of the young person–facing platform, clinicians emphasized the importance of visuals, compared with primary text, and features, such as earning badges and creating avatars, to keep young people engaged. Clinicians underscored the importance of designing the program brand to be discreet and having other privacy features programmed to ensure confidentiality of mental health information stored on devices. Clinicians unanimously agreed that using digital tools to augment therapy would be most effective if a human, such as a therapist, parent, or teacher, was behind this tool to check in with and guide young people. Another consideration discussed when using digital tools to augment therapy was the importance of setting up expectations and boundaries with the young person and parents so the capabilities of the tool can be understood by them. Clinicians also suggested working with the families so that it is understood that immediate help may not be available via this modality in crisis situations and to create a safety plan for those instances. Another strategy mentioned to mitigate the risk of an unrecognized crisis communicated via digital devices was to program the tool to automatically detect and guide the young person to the appropriate contact and resources.	

^a^DMH: digital mental health.

### Theme 1: Digital Tools in Clinical Care With Young People

#### Overview

Given the rapid emergence of technology, with young people at the forefront, clinicians discussed how using digital tools in practice was a good way to connect with this population and meet them where they are. As a clinician stated:

The world we live in is technology driven, and the kids are way more technology savvier than we are.

In home 2

Clinicians discussed that the overwhelming majority of their clients preferred digital platforms to nondigital platforms:

And even a lot of clients, whenever I bring up anything about journaling or anything like that, they’re completely against writing things on paper. But they’ll do their notes on their phone.

In home 2

As such, clinicians reported that using this modality was an excellent way to better engage young people in treatment.

In the first report, Lattie et al [18] reported on clinicians using technological resources to support skill building and empowerment of adults and young people. The following section explores the particular types of digital tools clinicians reported using exclusively with young people, along with how they incorporated these tools in their service of this population.

#### Build Skills and Facilitate Learning

Most frequently, clinicians mentioned using digital tools to help children and adolescents acquire and practice skills in treatment sessions, with the intent of young people using these skills in their daily lives. Several clinicians, for example, used digital tools, such as apps and web-based videos, to help guide their child and adolescent clients through practice sessions focused on skills such as meditation, relaxation, and mindfulness. Clinicians modeled and practiced these techniques with children and adolescents in the session while also encouraging them to practice these skills in everyday life between sessions. As a clinician mentioned:

I’ve been doing a lot of imagery work and progressive muscle relaxation prompts. So, I’ve had some of my kids record on their phone their own voice using those prompts, so that they can use it before bed or when they get up in the morning. And then kind of a way to empower them too.

School 1

This clinician empowered young people by having them lead the relaxation exercise in a digital recording on their personal devices, which reinforced their knowledge and practice of that skill and also led to a newfound familiarity with tailored tools that they could use in their daily lives. Using gamification to support skills such as communication was also mentioned. Although not designed as a DMH tool, a clinician reported using the app *Heads Up*, where the device was placed on the forehead of one person, and the other person used word cues to have that person guess the word or phrase presented on the device screen. This clinician commented:

I’ve seen some kid clients come alive because they’re excited because they wanna beat their score. And just helping them like, “How do you have to communicate? You have to keep talking. You have to keep going.” It’s helped with that.

Outpatient 1

Apps such as these and others specifically designed as DMH tools were used not only to engage young people in the treatment session but also to prompt practice of particular skills in real time.

Digital tools have also been used to demonstrate the value of therapeutic techniques. When wanting to provide tangible evidence of the mind-body connection to the young person, a clinician mentioned:

...since I got my Apple Watch, I’ve been letting kids put it on and then having them do jumping jacks and showing them their heart rate and then using the breathe app, so they see it. And they’re, “Oh, my gosh. My heartbeat went down when I took deep breaths.” And I was like, “Yeah. Your body’s calming down.”

School 1

The clinician used biofeedback via the Apple Watch to show the child how deep breathing helped slow down their heart rate. Similarly, other clinicians mentioned using different digital tools such as Fitbit to direct the child and adolescent clients through breathing exercises, whereas others mentioned apps, such as a drawing app or mediation app, to help the client downregulate emotions at the moment. After teaching and practicing the skills with young people in a session, clinicians mentioned encouraging them to practice the learned skills during the week (with or without the support of the demonstration technology) and equally encouraged parents to support their children in these efforts.

Clinicians also discussed using several different types of digital tools, such as web-based videos, websites, and apps, to facilitate learning and discussions during sessions. In particular, digital tools are frequently used to support psychoeducation around skills, such as mindfulness, or mental health conditions such as attention-deficit/hyperactivity disorder, which then prompted discussions with their clients. For example, several clinicians mentioned viewing YouTube videos tailored to their client’s needs together in session and then discussing the contents of the video, asking questions such as, “Did you understand this? What did you think about this?” [Outpatient 1].

Other clinicians mentioned using apps, such as interactive story apps where the user *chooses their own adventure* to provide tangible and engaging examples of situations to young people and then talking through the actions the child or adolescent chose in these apps. Overall, clinicians emphasized that digital tools provided a platform that initiated learning in a way that was engaging and understood by young people and also elicited rich discussions around key topics of interest. Clinicians mentioned that a unique benefit of using digital tools in practice was the ability to tailor the lessons to the specific mental health needs of the child or adolescent, as reported in Lattie et al [18] and their developmental stage (eg, Mind Yeti for adolescents with attention-deficit/hyperactivity disorder and GoNoodle for children with attention-deficit/hyperactivity disorder).

#### Monitor Symptoms

Other common digital tools that clinicians described using with young people were tracking tools, such as apps like Daylio, to chart symptoms between sessions. A clinician mentioned:

I use Moodpath, which helps clients especially–it’s for depression. So, tracking the symptoms. They can use the smiley faces throughout the day to track where they’re at.

Outpatient 1

The clinician further described that this app generated a chart that depicted the daily and weekly patterns of symptoms, which they found very valuable to reference periodically in session with the client. This resonated with other clinicians in the group, especially the utility of the tracking apps in engaging young people in real time throughout the week in language young people could understand and relate to (eg, smiley faces), compared with the complex, and often difficult to understand, clinical terms used to describe anxiety and depression symptoms. A clinician described how the mood-tracking app was used in session:

They [the adolescent] can bring it up on their phone...and we look at just is she daily fluctuating? If so, what happened during that day? What happened during that week? Is this a cycle? Is it three days anxiety, two days depression? Was it five days she had anxiety?

Outpatient 1

As depicted in this example, clinicians described using digital tracking tools as a platform to facilitate and tailor the conversation with young people by discussing patterns, precursors, and the context of their symptoms.

### Theme 2: Challenges of Using Digital Tools in Practice

#### Overview

Along with the benefits of using digital tools with young people, clinicians have also reported on their concerns. In the first report, Lattie et al [18] underscored that the major concerns clinicians had were around the confidentiality and privacy of digital tools. For example, concerns were expressed regarding confidentiality breaches if others, such as a friend or family member, used the client’s device or in situations in which the client used a communal digital device (eg, shared device among family or within a public setting). Additional barriers to the use of digital tools in clinical care are discussed as follows.

#### Accessibility

Several clinicians expressed accessibility concerns about adolescents’ use of digital tools outside of treatment. One such concern was broadband access, for example, a clinician said:

Because some kids don’t have data plans. They just have a phone. That they found. Their data goes fast so they’re bouncing off of Wi-Fi.

School 2

Indeed, challenges related to limited or no access to broadband and/or digital devices have been frequently mentioned. Another barrier was the frequent report of parents removing devices and internet access from their teenagers as a form of punishment. This was outlined by a clinician:

But then I’m thinking about the fact that it [young person’s smartphone] is normally one of the first things that get taken away if they do have a bad day. So, this is the thing you can use when you’re having a bad day to calm down, but then mom and dad won’t let you use it because you had a bad day, so you’re even more frustrated because you don't get to use the thing you’re supposed to use when you have a bad day.

School 2

Clinicians mentioned other restrictions caregivers and schools placed on device use, such as limits on screen time and the context in which the device can or cannot be used. For example, a clinician described:

My kids, they do guided meditation at home. Right before bed, to calm themselves or something like that. But that’s–a lot of parents have rules, like not past ten.

School 2

These restrictions, although typically implemented by parents to promote well-being, can significantly interfere with the ability of adolescents to engage in DMH programs when they perceive themselves most in need of the tools.

#### Outdated Programs and Limited Content

Other salient concerns of incorporating digital tools in their care for young people were the outdated design of many DMH tools and the limited content of other tools. Referring to an app designed to help maintain healthy habits, a clinician mentioned the following:

The clients that I got to try it, they would try it for a day and then they would be like, ‘Okay, I’m done with this.’ I wish that–it has a really great usage to it and everything, but it just needs to be updated.

In home 1

Although clinicians could see the utility of the app beyond its current outdated design, young people were often deterred from initially engaging or maintaining sustained use with these apps based on outdated design and content. Similarly, other digital tools that clinicians used had limited content, and thus their clients would become bored quickly or tire easily of these apps because they ran out of new content quickly.

### Theme 3: Desired Digital Platform and Integration Considerations

#### Centralized Digital Platform

Several clinicians desired a centralized digital tool that securely connected the clinician, young person, and young person’s family. As a clinician underscored:

I’d say I would find it really cool–again just thinking about my couple teenagers that use a mood tracking app–if there was some sort of way we could link accounts securely so that I could even login...Or even if I say, “Hey, do this activity on this app before I see you next week.” I can check and see when–like some sort of interaction base would be awesome.

In home 2

Clinicians viewed this type of tool to meet several goals. For example, they imagined that clinicians could use this tool to suggest apps or digital tools to young people and their families for use outside of sessions, remind young people to complete activities or practice skills between sessions, securely track their clients’ mood patterns and progress in real time, securely touch base with clients outside of treatment broadly and in specific situations (eg, weeks when the in-person sessions are canceled), and connect directly and securely with parents to check in and provide resources. They also mentioned that this tool could be used to better engage the parents in their child’s mental health care, with a clinician describing the following:

[I] feel like sometimes I’ll give parents follow up things to do while I’m not there, and they’ll forget about it throughout the week, but because they’re on their phone or whatever so much throughout the week, I feel like we could send them reminders or this is what we need to do before the next week. I think that that would encourage them to be more engaged, at least in the process.

In home 1

Clinicians also brainstormed their ideas for the young people–facing platform of this centralized tool and other young people–facing platforms. They discussed that this type of service would fit well within schools and other settings teens frequent and that they believe this modality would be preferable to a teen compared with paper worksheets that are easily lost, not interactive, or discreet. The teenager could, for instance, inconspicuously use their smartphone or school tablet when they are anxious or angry to practice learned strategies (eg, relaxation and cognitive restructuring). For example, a clinician suggested it would be:

some sort of app they could have on their phone that could help them. I think sometimes we teach them things, and they don’t remember to do them at home when they’re feeling upset. So, maybe it could be something positive that our kids could use when we’re not with them and we can’t review coping skills with them...

School 2

For this to be effective, however, clinicians underscored the importance of building rapport and attaining buy-in from parents and other stakeholders (eg, teachers) so that teens have access to these digital devices and services (eg, smartphones and internet access) to use as therapy resources when triggered at school or home.

Regarding the design of the young people–facing platform, clinicians emphasized the importance of visuals, compared with primary text and features, such as earning badges, to keep young people engaged. As a clinician reasoned:

They love badges. And decorating their avatars, like getting a new hat...So, they’re very motivated to get through their modules when they get to earn something at the end.

School 2

In addition to engagement, clinicians discussed the importance of privacy and being inconspicuous. For example, if the platform is on the young person’s device, having a password or facial recognition to get in and a subtle design if a peer or friend uses the teen’s device, they will not be immediately aware of the platform’s function, nor will they be able to easily gain access. As a clinician outlined:

The app doesn’t read as something like, My Personal Diary...it reads as something that you might just pass by if you don’t know what its intention is, which can be good for teenagers who are afraid of people looking into their stuff.

School 1

#### Considerations for Integration Into Care

Clinicians unanimously agreed that such a tool would be most effective if a human, such as a therapist, parent, or guidance counselor, were *behind* this tool to check in with and guide young people. They talked about who would be the best person to support the teen via this service and emphasized training that person. Specifically, training that person not only on the technical side of the mental health platform but also around safeguards and ethical considerations when using this platform with young people and families. They also mentioned the importance of setting up expectations and boundaries with their clients and parents in the context of this tool, so the capabilities of the tool can be understood by them. As a clinician stated:

So, you set up the boundaries at the beginning...“This is what this can help you with. This is what it can’t. This is when we really need to have that face to face.”

School 1

By doing so, young people and their families can fully use all the service’s functions and also understand that immediate help may not be available via this modality in a crisis situation. This safeguard was discussed as a means to mitigate the risk of an unrecognized crisis, in addition to other strategies, such as programming the tool to automatically detect and guide the teen to the appropriate contact and resources.

## Discussion

### Principal Findings

Given the rapid emergence of technology use among young people [7,8,22], clinicians have expressed great interest in more effectively incorporating technology into their clinical care with young people. Similar to previous research [13], clinicians noted that, in general, using this modality was an excellent way to better engage young people in treatment, and in particular, offered a novel way to build skills, facilitate learning, and monitor symptoms. There was a notable variety of the types of tools that were used—from the use of a heart rate feature to provide *in vivo* biofeedback during in-session mindfulness exercises to the use of web-based videos to facilitate learning and discussions around the child’s specific mental health need in language that is relatable and age-appropriate to the use of mood-tracking apps to facilitate conversations around patterns and precursors to changes in teen’s mental health symptoms. Our findings extend the quantitative results of Cliffe et al [12] by providing a more nuanced view on how clinicians are using digital tools with young people.

At the same time, clinicians expressed concerns about young people’s limited or complete lack of access to digital devices and connectivity outside of treatment sessions. Their feedback provided a different view from recent reports indicating nearly ubiquitous teen access to technology [7]. Their feedback suggests that accessibility [13] is much more complex, including considerations around inconsistent access (eg, shared device with family), limited connectivity or data plans, and conditional restraints specific to young people (eg, device taken away as punishment and school restrictions). Barriers such as these pose a clear threat to the utility of digital tools to provide young people with mental health support in real time when triggered. It is critical to include caregivers and others who play a prominent role in young people’s lives, such as teachers, in the treatment plan so that barriers, such as technology restrictions, can be pre-emptively addressed. As for connectivity and device access, our results imply that solutions, such as making content available offline or providing desktop versions, should be considered so that accessibility is not dependent on consistent internet and device access. Similarly, DMH tools could be offered via more accessible technology, such as native apps that do not require a consistent Wi-Fi connection to function, or through SMS text messaging to allow for interventions to be delivered to individuals without smartphones. Finally, it is important to highlight that these accessibility concerns were reported by clinicians who primarily work with families of lower socioeconomic status. If we are to strive for health equity and digital inclusivity through ubiquitous access to DMH tools, it is imperative that we understand and address the barriers, such as those mentioned, faced by already underserved and marginalized populations [23]. Without such considerations, the introduction of inaccessible DMH tools could lead to an exacerbation of existing disparities and inequities, as opposed to mitigation [24].

There was high interest among clinicians in a centralized digital tool connecting therapists, young people, and their caregivers. Clinicians were especially attracted to the possibility of a centralized tool to help generalize skills learned in sessions to young people’s daily lives and increase parental communication and engagement in their child’s treatment. The involvement of a young person in a centralized tool will most likely depend on their age. For example, the clinician could connect with a teenager and parent via the tool; however, the clinician would likely only be interacting with the parent if the client was of a younger age. Furthermore, clinicians had several design recommendations for young people–facing platforms. To increase initial engagement and maintain sustained use, clinicians underscored the importance of keeping the design and content of young people–facing platforms fresh and up to date [25]. Features such as badges and avatars were underscored to combat the vast drop-off typically observed in DMH tool use [26]. Visuals were also recommended in contrast to lengthy texts and readings to capture and maintain children’s and adolescents’ interest in the tool. Finally, clinicians emphasized the importance of designing the program brand, such as an app icon on a device, to be discreet and have other privacy features programmed (eg, facial recognition or fingerprint) to ensure confidentiality of mental health information stored on devices. These privacy preferences are consistent with previous studies in which teens and their therapists prioritized ambiguous branding of an app (ie, MD vs Mood Diary) to keep curious siblings or friends from accessing their mental health information [14].

Strikingly, when discussing the use of DMH tools, clinicians overwhelmingly outlined how they used tools within their face-to-face sessions. In this way, DMH tools were integrated into care in bespoke ways, and clinicians, therefore, surfaced several considerations for broader implementation. First, they underscored the importance of including some level of human support with digital tools. The importance of human support aligns well with frameworks around technology-enabled services [4,27,28] and research that suggests digital tools are more effective for some users when supported by coaches than standalone tools [29]. This is also consistent with other research in which clinicians who work with adults recommend that DMH tools should be used to enhance face-to-face treatment, not as a replacement for it [30,31]. It is therefore critical that clinical training and continuing education keep pace with the increased interest and expectation to integrate digital tools in routine care by upskilling clinicians’ DMH literacy, providing training for digitally enhanced models of care. Second, clinicians emphasized the importance of setting boundaries with young people and families when augmenting therapy with digital tools, similar to previous literature [13]. They suggested working with families so that it is understood that immediate help may not be available via this modality in crisis situations and to create a safety plan for those instances. Clinicians also brainstormed that a response to this challenge could involve algorithms built into tools that detect crisis-related words or phrases and automatically connect users to resources and services [32].

Most of these recommendations can be easily incorporated into the design of future DMH tools; however, the challenge of successful DMH implementation is still significant. Achieving successful and sustainable integration of digital tools into a predigital health system will likely require collaboration between specialists in DMH and implementation [33-37] and a shift from randomized controlled trials to effectiveness-implementation hybrid trials [38]. Only then can the field build evidence on how DMH tools can be successfully embedded into the daily work of clinical settings and have continued success without research support. Critically, such research and testing of different implementation strategies have a high potential to help fill the research-to-practice gap and to create sustainable tools within care settings that fulfill the promise of DMH [38].

### Limitations and Future Directions

It is important to understand our findings in the context of their limitations. This study elicited feedback from clinicians and supervisors practicing in a large community behavioral health care organization. Although this is the first step in designing DMH tools that can be incorporated into mental health care for young people, it is critical that these findings are complemented by feedback from young people undergoing treatment. Young people provide unique feedback based on their lived experiences and their own use of DMH tools within care settings [14,39,40]. In addition, gathering feedback from other stakeholders (eg, caregivers and teachers) who play a prominent role in the lives of young people will also be significant in creating DMH tools that can be used and seamlessly incorporated into young people’s lives. Given the paucity of research on DMH tools for child and adolescent mental health providers [12-14], it will also be important to replicate or elicit feedback from clinicians from other health care settings serving diverse populations of young people. We can then begin to understand which features of DMH tools are universal and which features are most effective within particular settings and populations of young people and clinicians. Furthermore, it is important to note that two sets of research questions were pursued with the large data set garnered from the focus groups. The research team took particular precautions to verify the integrity of the data, such as partnering with a key stakeholder from the community behavioral health care organization to verify the credibility and an independent check of result overlap. As qualitative data collection and analysis gain traction in the field of DMH, it is important to establish standards of practice for the field to ensure rigor and credibility. Finally, a representative from the community behavioral health care organization reached out directly to the research group to learn more about incorporating technology into their organization, and there was interest expressed from organizational leadership that led to this series of focus groups. Thus, this particular group of clinicians may be more interested in DMH tools than other clinicians. It will be important to include feedback from additional behavioral health care settings and providers with varying interests in incorporating DMH into practice.

### Conclusions

This study examined feedback from child and adolescent mental health care providers from a large community mental health organization on the use of digital tools used in care settings. Clinicians discussed how they incorporated digital tools into their clinical care to enhance skill building, facilitate learning, and monitor symptoms. Clinicians also provided insight into accessibility, suggesting that considerations should include consistency in access, connectivity, and conditional restraints specific to young people. Finally, clinicians expressed high interest in a centralized digital tool to help consolidate learned skills in daily life and increase communication with parents. Future studies are needed, especially those that elicit feedback from young people and other stakeholders, to form a body of literature that guides the design and implementation of sustainable DMH tools that support the mental health of children and adolescents.

## Abbreviations

ACTS: Accelerated Creation-to-Sustainment
DMH: digital mental health
